# Immunomodulatory Activity *in vitro* and *in vivo* of Polysaccharides from Kabuli Chickpea (*Cicer arietinum* L.) Hull

**DOI:** 10.17113/ftb.58.04.20.6634

**Published:** 2020-12

**Authors:** Hafiz Muhammad Saleem Akhtar, Zipeng Ye, Mohamed Abdin, Yahya Saud Hamed, Guijie Chen, Xiaoxiong Zeng

**Affiliations:** College of Food Science and Technology, Nanjing Agricultural University, Weigang 1, 210095 Nanjing, PR China

**Keywords:** *Cicer arietinum* L. hull, polysaccharide fractions, immunomodulatory activity, *in vitro* and *in vivo* activity, functional food

## Abstract

**Research background:**

Polysaccharides isolated from plants, fungi and bacteria are associated with immunomodulatory effects. Chickpea hull, which is regarded as food industrial waste, contains considerable amount of antioxidants and bioactive compounds.

**Experimental approach:**

In the present study, we investigated the immunomodulatory activity of polysaccharides from kabuli chickpea (*Cicer arietinum* L.) hull (CHPS). *In vitro* study was conducted with RAW264.7 cell line while *in vivo* study was carried out using specific pathogen-free BALB/c mouse animal model.

**Results and discussion:**

In *in vitro* test with RAW264.7 murine macrophage cells, the three purified fractions of chickpea hull polysaccharides showed potent immunomodulatory activity. Sample CHPS-3 showed stronger effect on cell viability, promoted the phagocytosis index to a greater extent and had the best effect on acid phosphatase activity. Moreover, it was found that CHPS-3 significantly (p<0.05) enhanced the secretion of nitrogen monoxide and cytokine (interleukins IL-6, IL-1β and tumor necrosis factor-alpha (TNF-α)) levels. In *in vivo* study, CHPS-3 improved thymus and spleen indices in cyclophosphamide-induced immunodeficient mice. Increased activities of lysozyme, catalase, superoxide dismutase and glutathione peroxidase, serum haemolysin content and total antioxidant capacity were observed, while the amount of malondialdehyde in the liver decreased.

**Novelty and scientific contribution:**

The results suggest that chickpea hull polysaccharides enhanced the immune activity and could be developed as the ingredient of functional foods.

## INTRODUCTION

Pulses have been part of human diet for thousands of years. However, the interest in and potential dietary impact of pulses on human health have only been revived during the past two to three decades. Chickpea (*Cicer arietinum* L.) is pivotal pulse crop produced and utilized globally, especially in African and Asian countries. It has been reported to be produced in more than 45 countries, ranking the third important pulse crop in the world (*1*, *2*). From economic point of view, kabuli chickpea is considered more important than desi chickpea because it gets higher market price (*3*, *4*).

Chickpea contains many bioactive compounds such as oligosaccharides, enzyme inhibitors, phenolic compounds and phytates that could assist in reducing the chronic disease risk. It serves as a worthwhile source of nutrients in vegetarian diets, especially in developing countries. Moreover, chickpea is rich in fibre and low in fat (*5*, *6*), being important source for a balanced diet. Some important vitamins are found in chickpea such as folate, thiamin, niacin, riboflavin, β-carotene and vitamin A precursor (*4*). Chickpea consumption has numerous physiological health advantages and in combination with other cereals and pulses, it has favourable effects on alleviating the symptoms of some major human diseases, such as digestive disorder, cardiovascular disease, type 2 diabetes and cancer (*2*, *6*).

Polysaccharides are high-molecular-mass polymers, which can be found as storage carbohydrates (starch) or as structural carbohydrates (cellulose). Natural polysaccharides are widely distributed in microorganisms and plants. Some of them have been confirmed to have various bioactive properties, such as antioxidative, antifatigue, immunomodulatory, antiatherosclerotic and hepatoprotective activities (*7*). Furthermore, polysaccharides extracted from yeasts, fungi, plants and algae have been increasingly utilized in food, biochemistry and pharmacology because of their wide range of biological functions (*8*).

There has been interest in recent decades in using polysaccharides as ideal immunomodulators (potential therapeutic agents) that can act by inducing the immune response of macrophages (*7*, *9*). Research shows excellent biological functions and nutritional value of natural polysaccharides from legumes and cereals (*9*-*11*). However, the information on the immunomodulatory activity of polysaccharides from chickpea hull is unavailable so far. In our prior study, we investigated the chickpea hull polysaccharide (CHPS) extraction optimization, characterization and *in vitro* antioxidant activities (*12*). In present study, therefore, first we determined the *in vitro* immunomodulation of CHPS using RAW264.7 murine macrophages, and then performed *in vivo* study using specific pathogen-free (SPF) BALB/c mouse animal model.

## MATERIALS AND METHODS

### Materials

The kabuli chickpea seeds were acquired from Urumchi (local market), Xinjiang Uygur Autonomous Region of China. Lipopolysaccharide (LPS), penicilin, streptomycin and 3-(4,5-dimethylthiazol-2-yl)-2,5-diphenyltetrazoliumbromide (MTT) were purchased from Shanghai Bioengineering Co., Ltd (Shanghai, PR China). Trichloroacetic acid, bovine serum albumin and dimethyl sulphoxide (DMSO) were procured from Solarbio Co. (Beijing, PR China). Guinea pig serum and 2% sheep red blood cell (SRBC) were obtained from Shanghai Chuanxiang Biological Technology Co., Ltd. (Shanghai, PR China). Cyclophosphamide (Cy) and levamisole hydrochloride were purchased from Shanghai Ruiyong Biological Technology Co. Ltd. (Shanghai, PR China). Assay kits for lysozyme, superoxide dismutase (SOD), catalase (CAT), glutathione peroxidase (GSH-Px), total antioxidant capacity (T-AOC) and malondialdehyde (MDA) were bought from Nanjing Jiancheng Bioengineering Institute (Nanjing, PR China). Dulbecco’s modified Eagle complete medium (DMEM) 1640 and newborn calf serum and Triton X-100 were obtained from Gibco/Invitrogen (Carlsbad, CA, USA). RAW264.7 cell line was arranged from Nanjing Keygen Company (Nanjing, PR China). Specific pathogen-free (SPF) grade BALB/c mice were purchased from Beijing Weitong Lihua Experimental Technology Co., Ltd. (Beijing, PR China). Nitrogen monoxide (NO) kit was obtained from Beyotime Institute of Biotechnology (Shanghai, PR China), while enzyme-linked immunosorbent assay (ELISA) kits for cytokines (interleukins IL-6, IL-1β and tumor necrosis factor-alpha (TNF-α)) were bought from Neobioscience Technology Company (Shenzhen, PR China). Sephadex G-100 column and neutral red were procured from Sigma-Aldrich Chemical Co., Merck (St. Louis, MO, USA). All other used chemicals were of analytical grade.

### Preparation of chickpea hull polysaccharides

The CHPS were prepared as per the reported procedure of Ye *et al.* (*12*). Briefly, chickpea hull (206 g) was separated manually from chickpea seeds (6 kg) with a yield of 3.43%. The hulls were soaked in water (at room temperature) for 4-5 min and then removed with tweezers. The hull was ground to powder, sieved through 60 mesh and extracted with hot water. The extract was then centrifuged at 3260×g (centrifuge model TDZW-S; Changsha Xiangzhi Centrifuge Instruments Co., Changsha, PR China) for 15 min. The resulting supernatant was concentrated, mixed with four volumes of absolute ethanol and kept overnight at 4 °C. The precipitates were centrifuged at 3260×*g* for 15 min, washed twice with dehydrated ethanol and lyophilized, obtaining crude CHPS. For purification, the crude CHPS was dissolved in distilled water, then loaded to DEAE Fast Flow column (Sigma-Aldrich, Merck, Chicago, IL, USA) and stepwise eluted with 0, 0.1, 0.3 and 0.5 M NaCl solution according to Ye *et al.* (*12*). The fractions were collected and the absorbance (*A*) was determined at 490 nm using the phenol-sulfuric acid method (*13*). Three fractions having carbohydrates were collected, concentrated, dialyzed, lyophilized and then further purification was done with Sephadex G-100 column (Sigma Chemical Co., Merck, St. Louis, MO, USA) chromatography by eluting with deionized water, respectively. The resulting fractions detected as mentioned above were accumulated, concentrated, then dialyzed and lyophilized, respectively, to obtain CHPS-1, CHPS-2 and CHPS-3.

### Immunomodulatory activity assay in vitro of chickpea hull polysaccharide

#### RAW264.7 cell culture

RAW264.7 cells were taken out of the liquid nitrogen tank and immediately thawed at 37 °C in the water bath. The cells were then transferred to a tube containing 5 mL of DMEM and centrifuged at 405×*g* (TDZW-S; Changsha Xiangzhi Centrifuge Instruments Co, Ltd.) for 5 min. The supernatant was removed, and the cell sediment was suspended with 1 mL medium containing 10% (*V*/*V*) newborn calf serum and 1% (*V*/*V*) penicillin-streptomycin mixture, transferred to a flat bottom culture box containing 3 mL of medium and incubated at 37°C in a humidified atmosphere of 5% CO_2_. When the cell growth reached 70-80% of the bottom of culture box, subculture was carried out.

#### Cell viability assay

RAW264.7 cell viability was measured in accordance with MTT-based colourimetric method (*14*) with minor changes. The cells were cultured on a 96-well culture plate (200 µL per well) at a density of 10^5^ cells/mL for 12 h. The non-adherent cells from the wells were aspirated by washing three times with phosphate-buffered saline (PBS) without cell rupture, and 100 µL of the medium were added to each well containing different concentrations (20.0, 40.0, 80.0, 160.0 and 320.0 µg/mL) of CHPS. LPS (10.0 µg/mL) and medium alone were used as positive and blank control, respectively. The cells were incubated for 24, 36 and 48 h. After incubation, 200 µL of MTT solution (0.5 mg/mL) were added to each well of the 96-well plate and further incubated for 4 h at 37 °C. Finally, the MTT solution was taken out, and 150 µL of DMSO were added to each well to terminate the reaction and dissolution of formazan crystals. After shaking for 5 min, the absorbance (*A*) at 570 nm was measured using a microplate reader (model 800 TS; BioTek Instruments Inc., Agilent, Winooski, VT, USA). Cell viability rate was measured as a ratio of *A*_sample_ and *A*_blank control._

#### Phagocytosis assay

Phagocytosis index of RAW264.7 was measured based on the findings of Zhang *et al*. (*15*), with some modifications. RAW264.7 cells were cultured as previously mentioned. CHPS solutions of different concentrations (20.0, 40.0, 80.0, 160.0 and 320.0 µg/mL) as well as a blank control (medium) and positive control (*γ*(LPS)=10.0 µg/mL) were added to a 96-well plate, and the plate was incubated at 37 °C in an incubator set at 5% CO_2_ for 24 h. After removing the medium, a neutral red solution (100 µL) was added to each well. After 1 h of incubation, the cells were washed twice with PBS to remove the excess neutral red. After that, a mixture of 100 µL of cell lysate (0.1 M acetic acid/ethanol=1:1) was added to each well and the plate was incubated again overnight at 37 °C. The absorbance was measured at 540 nm with microplate reader (model 800 TS; BioTek Instruments Inc.). The phagocytosis index was calculated as a ratio of *A*_sample_ and *A*_blank control_.

### Acid phosphatase activity

CHPS effect on acid phosphatase activity of RAW264.7 cells was determined as per previously reported method (*16*). The cultured cells (RAW264.7) were treated with complete medium alone or CHPS solutions (20.0, 40.0, 80.0, 160.0 and 320.0 µg/mL) or LPS (10.0 µg/mL). The supernatant was removed from the wells after incubation for 48 h at 37 °C. After that, 25 µL of 1% Triton X-100 and 150 µL of 1.0 mg/mL *p*-nitrophenyl phosphate were added into each well, and then the plate was incubated for 1 h at 37 °C. Then, 50 µL of 3.0 M NaOH solution were added to terminate the reaction. The absorbance at 405 nm was measured by a microplate reader (model 800 TS; BioTek Instruments Inc.). Acid phosphatase activity was measured as a ratio of *A*_sample_ and *A*_blank control._

### Nitrogen monoxide and cytokine assays

The colourimetric assay with Griess reagent was used to measure the released level of nitrogen monoxide in the medium (*14*). In a word, RAW264.7 cells (10^6^ cells/mL) were cultured for 24 h in 96-well plates with different concentrations of CHPS-3 (20.0, 40.0, 80.0, 160.0, 320.0 µg/mL), blank control (medium) and positive control (*γ*(LPS)=10.0 µg/mL). After that, 100 µL of culture supernatant were mixed with an equal volume of Griess reagent (1% sulfanilamide and 0.1% N-[1-naphthyl]ethylenendiamine dihydrochloride in 5% phosphoric acid). The samples were incubated for 15 min at room temperature. The absorbance was measured at 540 nm using a microplate reader (model 800 TS; BioTek Instruments Inc.). Standard curve was generated by using NaNO_2_. The cytokines (IL-6, IL-1β and TNF-α) were calculated by ELISA kit (Neobioscience Technology Company, Beijing, PR China) as per the manufacturer’s instructions.

### Immunomodulatory activity of CHPS-3 assay in vivo

In our previous study, we found that CHPS-3 had the strongest reducing power, highest scavenging activity against 2,2'-azinobis-(3-ethyl-benzothiazolin-6-sulfonic acid) and 1,1-diphenyl-2-picrylhydrazyl radicals, and prevention of H_2_O_2_-induced oxidative injury in PC12 cells (*12*). In the present study, CHPS-3 exhibited superior immunomodulatory activity *in vitro*. Hence, it was used for further study of immunomodulatory activity *in vivo*.

#### Animal grouping and experimental design

The experimental design followed the method by Liu *et al.* (*17*) with slight modifications. SPF BALB/c mice, 6-week-old with body mass (bm) of (20±2) g, were used. After a 7-day acclimatizing period, all mice were randomly divided into 6 groups (10 mice in each). The animal housing conditions were approved by the Ethical Committee of the College of Food Science and Technology, Nanjing Agricultural University, Nanjing, PR China, and strictly followed in accordance with the Chinese experimental animal management regulations. The temperature and relative humidity were maintained at (21±1) °C and 50-60% respectively. All mice were kept under standard maintenance conditions of 12 h light/dark cycle and were given free access to feed and water. Group I mice (normal control) were treated with 0.9% NaCl once a day for 17 days. Group II mice (model control) were treated with intraperitoneal injection of cyclophosphamide (Cy) administered at a dose of 80 mg/kg daily for the first 3 days of the experiment, followed by intraperitoneal injection of 0.9% NaCl for 14 days. Mice in group III (positive control) were treated with levamisole hydrochloride (dose 10 mg/kg b) instead of 0.9% NaCl administered in group II. Mice in groups IV, V and VI (low, medium and high-dose of CHPS-3 respectively) were treated with Cy for the first three days of experiment at the dose of 80 mg/kg followed by 50, 100 and 150 mg/kg bm of CHPS-3 per day for 14 days, respectively. After 12 h of the last feeding, all mice were sacrificed by cervical dislocation. During the experiment, it was essential to reduce the number of the used animals and their suffering.

#### Assay of thymus and spleen indices

The body mass of all the mice was measured in each group during the experiment. Moreover, thymus and spleen were removed and washed with normal saline to get rid of blood stains. The mass (*m*) of vital organs (thymus and spleen) was recorded. The mouse thymus or spleen indices (in mg/g) were calculated using the following equation:

Thymus or spleen index=(*m*(thymus or spleen)/*m*(body))∙100 /1/

### Serum haemolysin assay

On the tenth day of administration, the mice were injected with 0.2 mL of 2% sheep red blood cell (SRBC) suspension intraperitoneally. Twelve hours after the last administration, the serum was collected from the eye orbit of mice and normal saline was added for dilution. A volume of 200 µL of diluted serum, 200 µL of SRBC (2%) and 200 µL of guinea pig serum were added in sequence into a centrifugal tube. Furthermore, as a blank control the diluted serum was replaced with normal saline. The mixtures were placed in an incubator for 30 min at 37 °C and then centrifuged for 5 min at 6520×*g* (TDZW-S; Changsha Xiangzhi Centrifuge Instruments Co., Ltd.). The supernatant was collected and absorbance was measured at 540 nm (*A*_sample_) using a microplate reader (model 800 TS; BioTek Instruments Inc.). Moreover, the absorbance at which SRBC reached half the haemolytic condition (*A*_SRBC_) was determined. In short, 2% SRBC (1.0 mL) was centrifuged (600×*g*) with distilled water and then sediment was diluted to 950 µL. After resting for 10 min, NaCl solution (17%, 50 µL) was added to obtain haemoglobin solution 0.2%. The resulting (50 µL) haemoglobin solution, 50 µL of SRBC suspension and 400 µL of normal saline were added. Finally, after the centrifugation the absorbance of the supernatant at 540 nm was calculated as *A*_SRBC_. Serum haemolysin antibody content was expressed by HC_50_ as a ratio of *A*_sample_ and *A*_SRBC_.

### Serum lysozyme activity assay

The lysozyme activity of mice serum was determined by commercial kit (Sigma-Aldrich, Merck, Chicago) and protocol provided with the kit was followed. Lysozyme activity is defined as its dual activity as a lytic enzyme and a small cationic protein that damages or kills bacteria by lysing their cell wall peptidoglycan, by disrupting bacterial membranes, and by activating autolytic enzymes in the bacterial cell wall.

### Assay of antioxidant index in liver

After the mice were sacrificed, the liver was placed on ice and the tissue homogenate was prepared with physiological saline at a *m*/*V* ratio of 1:9. Centrifugation was done at 6520×*g* (TDZW-S; Changsha Xiangzhi Centrifuge Instruments Co., Ltd.) for 5 min, and the supernatant was collected for use. The levels of CAT, GSH-Px, T-AOC and SOD (U/mg), and MDA (nmol/mg) in mice liver protein were measured in accordance with the manufacturer’s instructions for the commercial kit (Sigma-Aldrich, Merck, Chicago).

### Statistical analysis

Data are expressed as mean values±standard deviation (S.D.). One-way ANOVA was carried out using Duncan’s multiple range test. Difference was considered to be statistically significant at p<0.05. SPSS software v. 19.0 (*18*) was used to perform the statistical analysis.

## RESULTS AND DISCUSSION

The crude chickpea hull polysaccharides (CHPS) were prepared from the kabuli chickpea hull and separated by column chromatography of DEAE Fast Flow and Sephadex G-100 according to the method reported by Ye *et al*. (*12*) with some modifications, giving three purified fractions of CHPS-1, CHPS-2 and CHPS-3. The fractions were used for the study of immunomodulatory activity.

### In vitro immunomodulatory activity of CHPS

#### Effect of CHPS on cell viability of RAW264.7

Cell viability assay depends on the potential of viable cells to metabolize a water-soluble tetrazolium salt into a water-insoluble formazan product. Macrophages are important immune cells in animals, with a number of immunomodulatory functions, such as engulfing foreign substances like antigens, and secretion of cytokines. Lipopolysaccharides (LPS), also known as endotoxins and lipoglycans, are large molecules comprising a polysaccharide and a lipid and they can be found in the outer membrane of Gram-negative bacteria (*19*). LPS as a proinflammatory factor which can initiate NO production in macrophages during the stimulation of i-NOS, and can induce cytokine expression (*20*). RAW264.7 is a mouse peritoneal macrophage and it is used for *in vitro* study of cell phagocytosis and cell immunity. In this study, the effects of CPHS-1, CHPS-2 and CHPS-3 on proliferation rate of RAW264.7 cells at different treatment times (24, 36 and 48 h) were measured by MTT method.

The results in Table 1 show that CHPS-1, CHPS-2 and CHPS-3 had a significant impact on the cell viability of RAW264.7 compared with control. At a concentration of 320 µg/mL and incubation time of 24 h, the significant effect of CHPS-3 on the growth of RAW264.7 cells was observed. The rate of cell viability was 2.4, which was significantly higher than that of the positive control under the same treatment conditions (p<0.05). When comparing different treatment times, incubation for 24 h generally had the best effect on proliferation of RAW264.7 cells. This might be due to the excessive amount of metabolites accumulated in the cell culture medium and less incubation time that might affect the cell growth. Similar effect of polysaccharides on cell viability was reported by Yuan *et al.* (*21*). It was found that polysaccharides from *Sinonovacula constricta* enhanced the RAW264.7 cell viability in a dose-dependent way from 12.5 to 100.0 µg/mL.

**Table 1:** Effects of chickpea hull polysaccharide fractions (CHPS-1, CHPS-2 and CHPS-3) on RAW264.7 cell viability

Sample	*γ*/(µg/mL)	*t*/h		
24	36	48
	Control	(1.00±0.02)^a^	(1.00±0.08)^a^	(1.00±0.05)^a^
	LPS	(1.6±0.3)^c^	(1.4±0.2)^cd^	(1.4±0.1)^e^
CHPS-1	20	(0.94±0.05)^a^	(1.14±0.05)^c^	(1.03±0.05)^c^
	40	(1.4±0.2)^b^	(1.4±0.3)^cd^	(1.24±0.08)^d^
	80	(1.3±0.2)^b^	(1.4±0.2)^cd^	(1.09±0.07)^c^
	160	(1.3±0.2)^b^	(1.2±0.2)^cd^	(0.9±0.1)^b^
	320	(1.0±0.2)^a^	(0.9±0.2)^a^	(0.70±0.06)^a^
CHPS-2	20	(1.32±0.09)^b^	(1.6±0.2)^c^	(1.1±0.1)^b^
	40	(1.47±0.06)^bc^	(1.7±0.2)^cd^	(1.2±0.2)^b^
	80	(1.6±0.1)^c^	(1.3±0.2)^b^	(1.17±0.05)^b^
	160	(1.81±0.09)^d^	(1.3±0.2)^b^	(1.14±0.06)^b^
	320	(1.4±0.1)^b^	(1.2±0.2)^b^	(1.2±0.1)^b^
CHPS-3	20	(1.4±0.2)^b^	(1.4±0.2)^b^	(1.45±0.1)^b^
	40	(1.6±0.2)^bc^	(1.5±0.2)^b^	(1.40±0.06)^b^
	80	(1.6±0.2)^bc^	(1.6±0.1)^b^	(1.4±0.1)^b^
	160	(1.9±0.2)^c^	(1.7±0.2)^bc^	(1.4±0.2)^b^
	320	(2.4±0.2)^d^	(1.9±0.2)^d^	(1.4±0.4)^b^

Values are presented as mean±S.D. (*N*=5) and evaluated by one-way ANOVA followed by the Duncan’s multiple range tests. Different letters in superscript denote significant difference (p<0.05). LPS=lipopolysaccharide

#### Effect of CHPS on phagocytosis index

The phagocytosis of macrophages plays a significant role in immune function. It has been reported that major cellular protection and manifestation of inflammatory and immunological responsiveness are due to the result of cell phagocytosis. When foreign antigenic substances enter into the body, these phagocytic macrophages use corresponding enzymes to dissolve these foreign particles. Hence, the phagocytosis of macrophages describes the first and determining phase in immune response (*22*). In this study, CHPS effect on phagocytosis of mouse peritoneal macrophages was evaluated by RAW264.7 cell with neutral red assay.

**Fig. 1:**
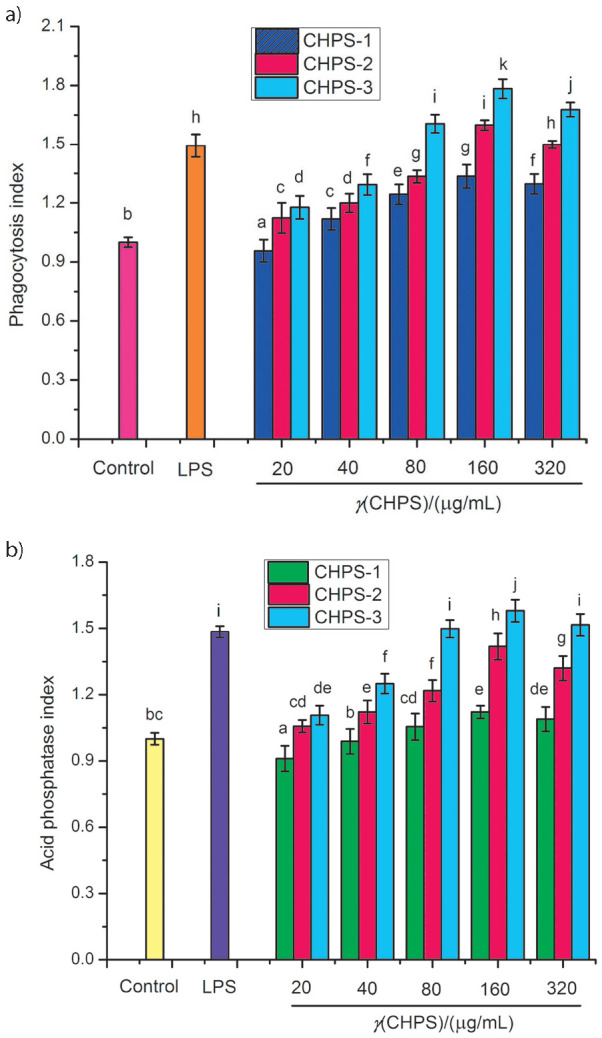
Effects of chickpea hull polysaccharide fractions CHPS-1, CHPS-2 and CHPS-3 on: a) phagocytosis index, and b) acid phosphatase activity of RW264.7. Values are presented as mean±S.D. (*N*=5), and different letters in each group denote significant difference (p<0.05). LPS=lipopolysaccharide

Fig. 1a shows that the phagocytosis indices of all three purified fractions of CHPS at different concentrations were greater than 1.0. The results indicated that all three purified fractions of CHPS had the ability to enhance phagocytic activity. Furthermore, it was observed that the promoting effects increased with the concentration of CHPS from 20 to 320 µg/mL. When the concentration reached 160 µg/mL, the phagocytosis index was the highest; however, it decreased at the concentration of 320 µg/mL. The findings are in accordance with a previous report which stated that verbascose from mung bean had different effects on the cell phagocytosis at different concentrations (*14*).

#### Effect of CHPS on acid phosphatase activity

Acid phosphatase is a signal enzyme for macrophage activation functioning as a lysosomal marker enzyme (*23*). Activation or inhibition of macrophages is directly associated with significant changes in acid phosphatase activity (*24*). The activity level of acid phosphatase can directly reflect the immunocompetence of macrophages. In the current study, the effects of CPHS-1, CHPS-2 and CHPS-3 on acid phosphatase activity of RAW264.7 cells are shown in Fig. 1b. The immune response increased with the increase of concentration of CHPS from 20 to 160 µg/mL, while it decreased at 320 µg/mL. Moreover, the acid phosphatase activity at 320 µg/mL of CHPS was significantly lower (p<0.05) than at 160 µg/mL. As reported, the polysaccharide from the peduncles of *Hovenia dulcis* had similar behaviour (*16*).

#### Effect of CHPS-3 on nitrogen monoxide and cytokine production

**Fig. 2:**
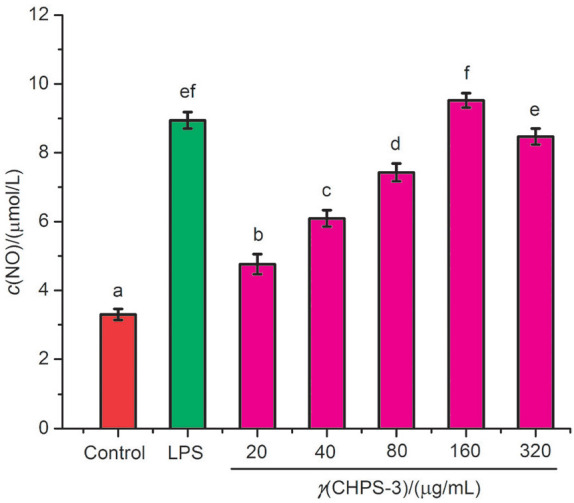
Effects of chickpea hull polysaccharide CHPS-3 on nitrogen monoxide (NO) production in RAW264.7 cells. Different letters represent a significant difference (p<0.05) between different concentrations of samples. LPS=lipopolysaccharide

NO is well known as neurotransmission modulator that protects against pathogens and act as vasorelaxant. Its release has been strongly related with immune response, inflammatory reaction, antiviral and antitumour activities (*25*). Thus, in the present study NO was considered as an index for evaluation of immunomodulatory effect of CHPS-3. Fig. 2 shows that NO secretion was significantly enhanced in RAW264.7 cells treated with CHPS-3 in a concentration-dependent manner, indicating the ability of polysaccharides to activate macrophage to release NO. The NO production increased with the increase of the concentration of CHPS from 20 to 160 µg/mL (Fig. 2). Greater increase in the production of NO by CHPS-3 is observable at the maximum concentration (160 µg/mL) than by the control. These results demonstrated that CHPS-3 is the most active immunomodulator with respect to NO production (p<0.05). The observations of the current study are in agreement with the results of Zhang *et al.* (*26*), where immunity response induced by polysaccharides of *Uncaria rhyncophylla* and *Taxillus chinensis* was observed.

**Fig. 3:**
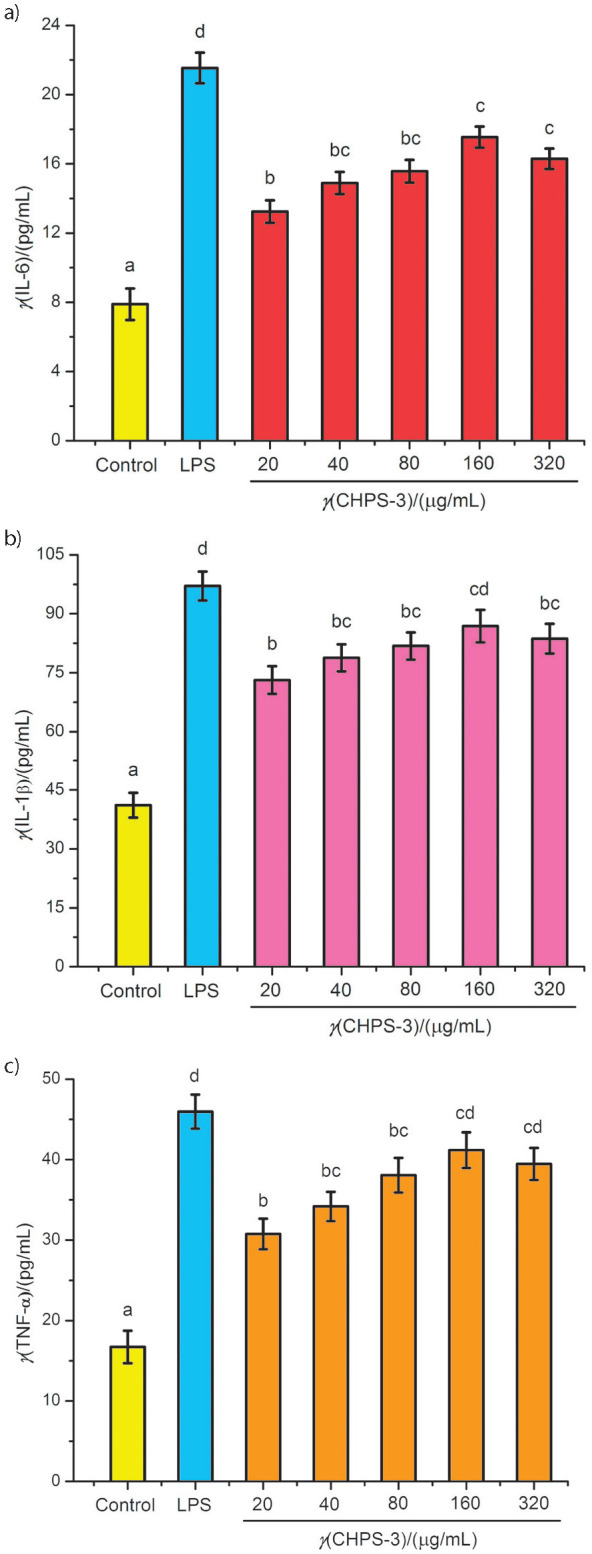
Effect of chickpea hull polysaccharide fraction CHPS-3 on the cytokine secretion in RAW264.7 cells: a) interleukin IL-6, b) IL-1β, and c) tumor necrosis factor-alpha (TNF-α). Different letters represent a significant difference (p<0.05) between different concentrations of samples

The immunological activity of polysaccharides in macrophages was further evaluated by the detection of cytokine (IL-6, IL-1β and TNF-α) production. Cytokines, produced by immune cells, are small protein molecules with anti-inflammatory and immunomodulatory activities (*27*, *28*). The known multifunctional prototypic cytokine is IL-1 (IL-1α and IL-1β), which influences every cell type primarily related with other cytokines. Leukocytes secrete IFN-α, which is predominantly linked to innate immune response against viral infection. IL-6 is linked with the phagocytosis, inflammatory regulation and antigen expression (*29*). Accordingly, in the present study, we investigated the effects of CHPS-3 on cytokine (IL-6, IL-1β and TNF-α) production. The cytokine (IL-6, IL-1β and TNF-α) production by RAW264.7 cells in all treatments with different concentrations of CHPS-3 was notably higher (p<0.05) than in normal control group (Fig. 3).

At concentrations of CHPS-3 between 20 and 160 μg/mL, the production of cytokines increased in a dose-dependent manner, while at concentrations between 160 and 320 μg/mL, there was no significant difference (p>0.05) in their production (Fig. 3). These results suggest that polysaccharides can boost immune response through the release of inflammatory mediators (NO) and immune cell factors (IL-6, IL-1β and TNF-α). It is well known that when macrophages are produced by bacterial toxins such as LPS, they engulf foreign matter and their proliferation is accelerated. At the same time, they secrete NO and cytokines as an immune response to hinder the growth of large agonistic substances. In a similar way to LPS, CHPS-3 also induced macrophages to secrete cytokines and NO. These defensive physiological reactions, in turn, could defend biological organisms against pathogens, cells damage or tumour cells (*30*, *31*). Similar results of cytokine production were reported in another study by Wang *et al.* (*32*), where immune response of polysaccharides from mushroom *Collybia radicata* was evaluated.

### In vivo immunomodulatory effects of CHPS-3

#### Effect of CHPS-3 on thymus and spleen indices

**Fig. 4:**
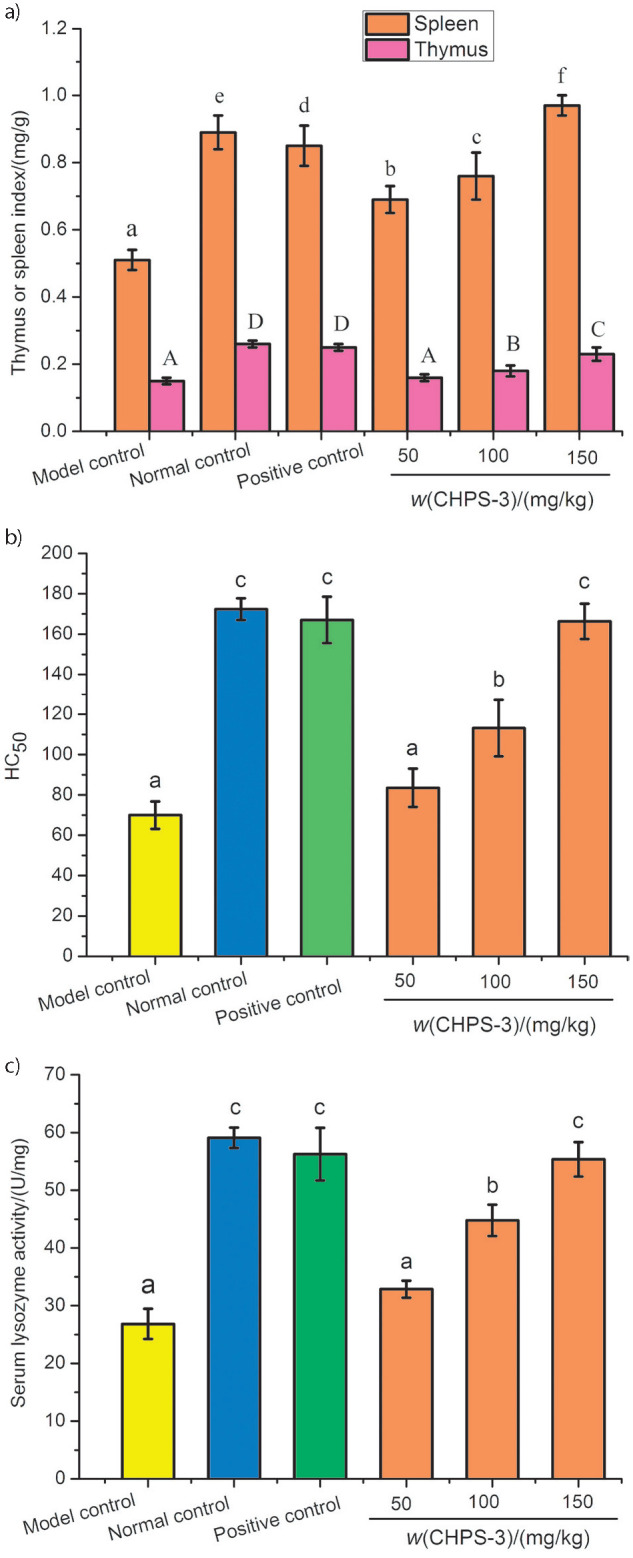
Effects of chickpea hull polysaccharide fraction CHPS-3 on: a) thymus and spleen indices, b) serum haemolysin (HC_50_) and c) serum lysozyme activity in immunosuppressed mice. Values are presented as mean±S.D. (*N*=3) and different letters in each group denote significant difference (p<0.05)

Spleen and thymus are important immune organs in animals. Thymus is the site of differentiation, development and maturation of T cells and is also related to the secretion of thymic hormone. Spleen contains a huge amount of macrophages and lymphocytes, which is the centre of cellular and humoral immunity in the body. The relative mass of the thymus and spleen can indirectly reveal the immune response of the body (*33*). In general, the relative mass of an immune organ in immunosuppressed individual is lower than in normal individuals. In this study, BALB/c mouse were immunosuppressed by injecting Cy. The thymus and spleen indices in normal, model and positive controls and CHPS-3 treatments are shown in Fig. 4a. Compared to the normal control group, the thymus and spleen indices in model control group decreased significantly, indicating that treatment with Cy damaged the immune organs and the immune system injury model was successfully established. After the administration of CHPS-3 for two weeks, however, the thymus and spleen indices in mice increased significantly and the spleen index of high dose group was higher than that of positive control. The results are in agreement with a previous report describing a significant difference in immune organ mass of mice treated with a low or high dose of polysaccharides from *Ganoderma atrum* (*34*).

#### Effect of CHPS-3 on serum haemolysin

Serum haemolysin plays an important role in humoral immunity, which is the immune response of immune factors such as antibody complement present in the body fluid. SRBC antibodies are produced by injecting SRBC into mice. Incubation with SRBC *in vivo* leads to haemolysis under the action of complement system. The measurement of absorbance indirectly reflects the content of haemolysin in the serum through which the amount of haemoglobin can be determined. Fig. 4b shows that the serum haemolysin content in the model control group was less than half of that in the normal control group, indicating that Cy could significantly reduce the serum haemolysin content. After the injection of levamisole hydrochloride, serum haemolysin level of the positive control group was restored to the level of the normal control group. When a low mass fraction of CHPS-3 was added, serum haemolysin level in mice was higher than that in model control group, but there was no notable difference observed (p>0.05). The serum haemolysin content in mice at CHPS-3 mass fractions of 100 or 150 mg/kg, however, was significantly higher than that of model control group (p<0.05). The hemolysin content in high dose group was close to that in normal control group (p>0.05). In the previous study, it was revealed that the administration of polysaccharides from *Cipangopaludina chinensis* at high concentration can significantly boost HC_50_ level compared with model group (p<0.05), proposing a distinctive stimulating action of polysaccharides on humoral immune response. It was described that several plant-based polysaccharides can enhance immune response by stimulation of B cells or generation of distinctive IgG, IgM and IgA (*35*).

#### Effect of CHPS-3 on serum lysozyme activity

Lysozyme, an alkaline protein containing four pairs of disulfide bonds, is widely found in animals, plants and microorganisms, and it is known to be effective innate immunity molecule (*36*). It can catalyze the hydrolysis of α-(1→4) glycosidic linkage between *N*-acetylmuramic acid and *N*-acetylglucosamine in the cell wall, thus alternating the sugar residues in the bacterial peptidoglycan, causing cell lysis of bacteria. Accordingly, lysozyme plays an important role in the body, such as anti-inflammation and humoral immunity. As shown in Fig. 4c, it is clear that serum lysozyme level in the model control group significantly decreased compared to that of normal control group (p<0.05). In the groups of mice treated with CHPS-3, there was no notable difference between the group administered low concentration and the model control group (p>0.05), however, the serum lysozyme content in the medium and groups administered high concentration significantly increased compared to that in the model control group (p<0.05). Serum lysozyme level in the group receiving high concentration was close to that in normal control group (p>0.05). These findings are similar to the report where polysaccharides from *Sophora subprosrate* significantly enhanced the serum lysozyme activity when mice were fed with it (*37*).

#### Effect of CHPS-3 on the antioxidant index in mice liver

It is well known that Cy can treat tumour and cause immune suppression, but it also causes side effects such as oxidative stress (*38*). To counter the oxidative stress problem, there are two kinds of naturally occurring antioxidant systems in the body, one is antioxidant enzymes and the other is small antioxidant molecules. Among them most important are antioxidant enzymes T-AOC, SOD, GSH-Px and CAT (*39*). The GSH-Px has the biochemical ability to reduce lipid hydroperoxides to their relevant alcohols, which then reduce free hydrogen peroxide to water, protecting the structural integrity of the cell membrane. T-AOC is the total antioxidant index, which can reflect the body's total antioxidant capacity. Thus, the antioxidant indices such as CAT, GSH-Px, T-AOC, SOD and MDA were determined in mice with Cy-induced immunodeficiency in this study.

As shown in Table 2, Cy could significantly reduce the activities of CAT, GSH-P_X_, T-AOC and SOD, and increase the content of MDA in the mice liver, indicating that Cy could cause oxidative stress in mice. Levamisole hydrochloride could restore T-AOC, GSH-Px, CAT and SOD activity and MDA content in mice to close to normal level (p>0.05). Low mass fraction of CHPS-3 increased the activities of CAT, GSH-Px, T-AOC and SOD in mice liver, and decreased MDA content, but did not increase significantly the activities of CAT and GSH-Px (p>0.05). With the increase of CHPS-3 mass fractions, T-AOC CAT, GSH-Px, and SOD activities in the liver of mice increased and the content of MDA decreased. When the mass fraction of CHPS-3 reached 150 mg/kg bm, the activities of GSH-Px, T-AOC, CAT and SOD and MDA content in the liver recovered to close to normal levels (p>0.05). From overall findings it was verified that CHPS-3 significantly improved the T-AOC, CAT, SOD and GSH-Px activities, but decreased MDA content in the liver, which is in accordance with reports for the polysaccharides from dietary litchi pulp (*40*) and *Sargassum fusiforme* (*41*).

**Table 2:** Effects of chickpea hull polysaccharide fraction CHPS-3 on antioxidant indices of immunosuppressed mice treated with cyclophosphamide (Cy)

Group	*w*(Cy)/(mg/kg)	Enzyme activity/(U/mg)				(*n*(MDA)/*m*(protein))/nmol/mg)
CAT	GSH-Px	T-AOC	SOD
Normal	-	(45.0±2.8)^c^	(321.2±16.8)^c^	(1.6±0.2)^c^	(133.7±1.9)^c^	(3.0±0.2)^ab^
Model	-	(21.9±6.8)^a^	(197.3±22.04)^a^	(0.6±0.07)^a^	(79.1±1.4)^a^	(5.6±0.6)^c^
Positive	10	(43.0±7.8)^bc^	(312.5±23.9)^c^	(1.6±0.08)^c^	(131.0±10.4)^c^	(2.3±0.6)^a^
CHPS-3 (low dose)	50	(25.7±2.4)^a^	(210.8±13.6)^a^	(0.9±0.05)^bc^	(106.5±5.1)^b^	(4.3±0.2)^bc^
CHPS-3 (medium dose)	100	(30.2±2.4)^ab^	(246.2±19.9)^ab^	(1.04±0.04)^b^	(114.1±1.8)^b^	(4.1±0.6)^b^
CHPS-3 (high dose)	150	(39.6±4.1)^bc^	(281.0±14.5)^bc^	(1.3±0.1)^c^	(121.5±7.6)^bc^	(3.5±0.8)^ab^

Data are presented as mean value±S.D. (*N*=3) and evaluated by one-way ANOVA followed by the Duncan’s multiple range test. Different letters in superscript denote significant difference (p<0.05). CAT=catalase, GSH-Px=glutathione peroxidase, T-AOC=total antioxidant capacity, SOD=superoxide dismutase, MDA=malondialdehyde

## CONCLUSIONS

In the present study, we demonstrated that the three purified fractions of chickpea hull polysaccharides (CHPS) could promote the cell proliferation rate, phagocytosis index and enhance the activity of acid phosphatase in murine macrophages of RAW264.7 *in vitro*. Among them, CHPS-3 exhibited superior immunomodulatory activity *in vitro*. Additionally, it was observable that CHPS-3 enhanced NO secretion and cytokine (IL-6, IL-1β and TNF-α) production. In the study *in vivo*, CHPS-3 improved the thymus and spleen indices in cyclophosphamide-induced immunodeficient mice. It increased the lysozyme activity, serum haemolysin content, CAT, GSH-Px, T-AOC and T-SOD activities, and reduced the content of MDA in the liver. In a word, CHPS exhibited strong antioxidant ability and immunomodulatory activity. Further studies to elucidate detailed mechanism of CHPS and its structure are in process.
